# Nutritional Status and Body Composition in Wilson Disease: A Cross-Sectional Study From China

**DOI:** 10.3389/fnut.2021.790520

**Published:** 2021-12-31

**Authors:** Hao Geng, Shijing Wang, Yan Jin, Nan Cheng, Bin Song, Shan Shu, Bo Li, Yongsheng Han, Yongzhu Han, Lishen Gao, Zenghui Ding, Yang Xu, Xun Wang, Zuchang Ma, Yining Sun

**Affiliations:** ^1^Laboratory of Sports and Nutrition Information Technology, Institute of Intelligent Machine, Hefei Institutes of Physical Science, Chinese Academy of Sciences (CAS), Hefei, China; ^2^Department of Biophysics, University of Science and Technology of China, Hefei, China; ^3^Hospital Affiliated to the Institute of Neurology, Anhui University of Chinese Medicine, Hefei, China

**Keywords:** Wilson's disease, nutritional status, body composition, NRS2002, phenotype

## Abstract

**Background:** Abnormal nutritional status is frequently seen in patients with chronic diseases. To date, no study has investigated the detailed characteristics of abnormal nutritional status among Wilson's disease (WD) patients in the Chinese cohort. This study aimed to describe the nutritional status of WD patients, with a particular focus on the differences between patients with different phenotypes.

**Methods:** The study subjects comprised 119 healthy controls, 129 inpatients (hepatic subtype, *n* = 34; neurological subtype, *n* = 95) who were being treated at the affiliated hospital of the Institute of Neurology, Anhui University of Chinese Medicine. All of the subjects were assessed for body composition by using bioelectrical impedance analysis. All WD patients received anthropometry, nutritional risk screening 2002 (NRS2002), and laboratory test (hemocyte and serum biomarkers) additionally.

**Results:** Compared with healthy controls, the fat mass and rate of total body and trunk were significantly higher in WD patients (*P* < 0.001), the muscle and skeletal muscle mass of total body and trunk were significantly lower in WD patients (*P* < 0.001). Compared with hepatic subtype patients, the fat mass and rate of total body, trunk, and limbs were significantly lower in neurological subtype patients (P<0.01); while there were no significant differences in muscle and skeletal muscle between these two subtypes. The overall prevalence of abnormal nutritional status in WD patients was 43.41% (56/129). The prevalence of high-nutritional risk and overweight in WD patients was 17.83% (23 of 129) and 25.58% (33 of 129), respectively. Compare with patients with high nutritional risk, macro platelet ratio, alkaline phosphatase, the basal metabolic rate (*p* < 0.05), creatinine, trunk fat rate (*p* < 0.01) and appendicular skeletal muscle mass (*p* < 0.001) were significantly higher in patients without nutritional risk (*p* < 0.001). Patients with a high nutritional risk tend to have a lower cholinesterase concentration (*x*^2^ = 4.227, *p* < 0.05).

**Conclusion:** Both patients with H-subtype and N-subtype are prone to have an abnormal nutritional status. Longitudinal studies are required to investigate if nutritional status and body composition could reflect prognosis in WD patients, and which of these body composition indexes contribute to malnutrition and worse prognosis.

## Introduction

Wilson's disease (WD) is an autosomal recessive copper metabolic disorder characterized by dysfunction of the liver, brain, bone and endocrine system mainly (1). The phenotype can be divided into hepatic subtype (H-subtype) and neurological subtype (N-subtype) according to the primary symptom (2). The prevalence of WD in China was 5.87 in 100, 000, which was higher than western descent (1 in 30,000) (3, 4). WD is potentially treatable, unlike most genetic diseases, and the life expectancy of some patients is about the same as that of normal people if they receive timely and correct treatment. However, there are still quite a few WD patients who have a poor prognosis for the reason of some avoidable factors, including incorrect diet, untimely treatment and irregular medication, etc. (5). Additionally, more unknown factors have not been found and the deterioration of some WD patients cannot be prevented and explained. Therefore, exploring and getting avoid of the controllable factors that may deteriorate the prognosis is being one of the most significant therapeutic targets concerned by neurology physicians. WD is a multisystem disease, which causes injury in not only the brain and liver, but also in renal, cardiac, skin, osteoarticular, or endocrinologic and includes other organ disturbances (6). This may influence the whole body's nutritional status. Additionally, lipid metabolism dysregulation is related to WD (7), which may affect the body composition of patients.

Nutritional status might be a potential factor that influences the prognosis of patients with WD. Abnormal nutritional status can be divided into nutritional deficiency (high nutritional risk) and nutritional overload (overweight) (8). Nutritional status is affected in subjects with Wilson's disease *via* many mechanisms such as the impact of long-lived chronic hepatic damage, renal injury, chelating medications, copper-restricted diet, imbalance of gut flora and dysphagia among patients with severe neurological dysfunction. These factors largely affect the intake, absorption and metabolism of nutrients from daily food. Body composition is another index that can reflect the nutritional status of patients with WD (9). It contains mass and rate of fat, muscle, bone, water and protein. The nutritional status was potentially ameliorated and frequently under-recognized, while it could play a crucial part in WD patients' long-term prognosis.

Nutritional status might be a potential factor that influences the prognosis of patients with WD. It has been proved to play an essential part in Parkinson's disease (PD), Alzheimer's disease (AD), and other chronic diseases. Cova et al. (10) utilized bioelectrical impedance measurements, nutritional scales (nutritional risk screening 2002, NRS2002; mini nutritional assessment, MNA) to explore the differences in nutritional status indexes among healthy individuals, mild cognitive impairment (MCI) subjects and AD patients. They found nutritional status indexes could characterize the process from normal to MCI to AD. Thus, the above nutritional status indicator set could potentially be a non-invasive, convenient, and trackable monitoring tool in the assessment, prevention, and efficacy evaluation of AD. Petroni et al. (11) assessed the nutritional status of patients with advanced-stage Parkinson's disease with body composition analysis, anthropometric measurements and serum biochemical markers. Their results demonstrated that obesity could be common among patients with advanced-stage Parkinson's disease, besides, fat-free mass and muscle mass were continuously consumed during the disease course. Lin et al. (12) reported the correlation between the nutritional status with the severity of PD symptoms. Those patients with poor nutritional status might have a worse prognosis. Weight, body mass index, hemoglobin, and cholesterol can be regarded as regular biomarkers to reflect the nutritional status and disease progression among PD patients.

There appears to be a paucity of medical literature with regard to the prevalence, magnitude, and feature of these nutritional manifestations in Wilson's disease. In this study, we aim to explore the characteristics of nutritional status in patients with WD, with a special focus on the relationship between nutritional status and clinical phenotype.

## Materials and Methods

### Subject and Design

This cross-sectional study was investigated by Sports & Nutrition Information Technology Laboratory, Chinese Academy of Science, and it was carried out in the Center of WD, the affiliated hospital of the Institute of Neurology, Anhui University of Chinese Medicine.

The study protocol was approved by the Ethics Committee of Hefei Institutes of Physical Science, Chinese Academy of Sciences (SWYX-Y-2021-08), and informed written consent from all subjects was obtained by the principal researcher, after self-motivated behavior evaluation of the patients' capacity to provide consent. In those patients under 18-year-old, their assent form was signed by their parents.

We enrolled a total of inpatients admitted from June 2020 to June 2021 with the diagnosis of WD (follow by the standard guidelines developed by the American Association of the Study of Liver Diseases). Healthy controls (HC) were recruited in the same period from communities in Anhui province.

Individuals were excluded if aged >55 years if they had pacemakers, heart defibrillators, bone nails, or other metal/electrical implants and if they were participating in exercise or nutritional intervention programs. To avoid interference from other metabolic factors, patients suffering from ascites, hepatic encephalopathy, diabetes, and hyperthyroidism were excluded.

All study participants underwent an evaluation following a standardized protocol. Collected data included demographic characteristics, medical history, pharmacological history and Unified Wilson's Disease Rating Scale (UWDRS) assessment.

### Nutritional Evaluation

The nutritional evaluation was performed using bioelectrical impedance analysis, anthropometry, nutritional risk screening 2002 (NRS2002), and blood test (blood routine examination and serum biochemical test).

Bioelectrical impedance measurements were conducted by a trained investigator and the participants ought to be fasted for at least 3 h. The bioelectrical resistance (R, Ohm) and body composition indexes (mass and rate of fat, muscle, bone, protein, water, etc.) were detected by an eight-electrode impedance analyzer (BX-BCA-100, Broshare Technology, Hefei, China). The accuracy of the body composition model was corrected using the standard of Tanita-980 (Tanita, Tokyo, Japan).

Anthropometric measurements included height, weight, body mass index (BMI), body circumferences (Waist, hip, biceps, mid-arm muscle, calf), skinfold thickness (triceps and subscapular) it was operated by the following standard criteria. All study participants were required to wear light clothing without shoes and socks. The room temperature and humidity were controlled at 24°C and 40%, respectively. Height (cm, to the nearest 1 cm) was measured with an altimeter and weight (kg, to the nearest 0.01 kg) with a standard scale; body mass index (BMI) (kg/m^2^) was hence calculated. Body circumferences were obtained with an inelastic plastic-fiber tape measure (to the nearest 0.1 cm); The waist circumferences were measured midpoint between the lowest rib and the upper border of the iliac crest; the hip circumferences were measured at the horizontal section between the pubic symphysis and the most convex part of the back gluteus at the maximum; the calf, biceps and mid-arm muscle were measured at the maximum girth. The skinfold thickness of triceps and subscapular was measured by a skinfold thickness gauge.

NRS2002 is recommended by the ESPEN guidelines and it takes into account the severity of disease and impaired nutritional status (13). In our study, every NRS2002 scale was conducted by an experienced specialist nurse and it should be re-examined by an attending doctor. Impaired nutritional status contains unexplained weight loss, reduced food intake, and BMI. Patients would get a score of 1 if their weight loss >5% in the last 3 months or 0 to 25% reduced food intake of the normal requirement; a score of 2 if weight loss >5% in the last 2 months, BMI was 18.5 to 20.5 kg/m^2^ plus impaired general condition, or 25 to 50% reduced food intake of the normal requirement; and a score of 3 if weight loss >5% in the last months, BMI of <18.5 kg/m^2^ plus impaired general condition, or 50 to 75% reduced food intake of the normal requirement. The final score of the NRS2002 ranged from 0 to 7, with a score of ≥3 indicating a high nutritional risk.

Blood routine examinations, including red blood cells, leukocytes, hemoglobin, platelets, were measured using a method of fluorescence flow cytometry with an automatic blood analyzer (SYSMEX, XT-1800i). Serum biochemical tests, including aminotransferase, bilirubin, monoamine oxidase, were measured using a colorimetric method with an automatic biochemical analyzer (HITACHI, 7600).

### Statistical Analysis

The characteristics of the subjects were described. Continuous variables were presented as mean values and standard deviations, and categorical variables were presented as counts. To compare the malnutrition status, data for NRS2002 were dichotomized into those at moderate or high risk (≥3) and those at a normal level (<3). Subjects with BMI ≥ 25 were defined as overweight. Men with a body fat rate >21.4% and women with a body fat rate >29% were defined as having a high body fat percentage (14). People with high nutritional risk or overweight were defined as having an abnormal nutritional status.

Continuous variables of characteristics among the three groups of participants (different subtypes and nutritional status) were compared using the univariate ANOVA test and student's test. Categorical variables of characteristics between the two groups (H-subtype WD patients and N-subtype WD patients) were compared using the Pearson chi-square test or Fisher's exact probability method. All statistical analyses were conducted using SPSS software (IBM SPSS Statistics Version 17.0). Statistical significance was set at *p* < 0.05.

## Results

The 129 WD patients were characterized based on four parameters: bioelectrical impedance analysis (BIA), anthropometry, nutritional status, and serum biochemical biomarkers. HC, patients with H-subtype and patients with N-subtype were 129, 34, and 95 cases each. The mean age of HC, patients with H-subtype, and patients with N-subtype were 28.17 ± 7.56, 28.86 ± 8.47, 25.2 ± 7.36, and 30.15 ± 8.49, respectively. No statistically significant difference in BMI had been found among these three groups. There were no statistically significant differences in gender composition ratio (*x*^2^ = 2.058, *p* > 0.05) between H-subtype WD patients (19 males and 15 females) and N-subtype WD patients (66 males and 29 females). Patients with H-subtype were all in the stage of liver fibrosis or compensated stage of cirrhosis, none of them had decompensated cirrhosis with ascites.

### The BIA Characteristics of Different Subtypes of WD Patients

Table 1 shows bioelectrical variables in healthy controls (HC), H-subtype Wilson's disease patients and N-subtype patients. No statistically significant differences in BMI between HC and patients with WD. The body composition was characterized by fat and muscle mainly.

**Table 1:** Bioelectrical variables in healthy controls and patients with WD.

	**HC** **(***n*** = 119)**	**Overall WD** **(***n*** = 129)**	***T*** **-value (HC vs. WD)**	**H subtype** **(***n*** = 34)**	**N subtype** **(***n*** = 95)**	***F*** **-value (HC vs. H subtype vs. N subtype)**	***T*** **-value (HC vs. N subtype)**	***T*** **-value (H subtype vs. N subtype)**
Age (y)	28.17 ± 7.56	28.86 ± 8.47	0.670	25.2 ± 7.36	30.15 ± 8.49	5.151^**^	1.813	−3.017^**^
Height (m)	1.73 ± 0.07	1.73 ± 0.09	0.162	1.75 ± 0.09	1.71 ± 0.08	3.167^*^	−0.82	2.19^*^
Weight (kg)	68.38 ± 12.13	68.08 ± 15.2	−0.172	73.18 ± 18.5	66.24 ± 13.46	3.248^*^	−1.215	2.005
BMI (kg/m^2^)	22.94 ± 3.34	22.78 ± 4.21	−0.346	23.7 ± 5.04	22.43 ± 3.83	1.457	−1.02	1.336
FFM (kg)	55.04 ± 9.29	51.11 ± 10.28	−3.155^**^	52.01 ± 12.58	50.77 ± 9.37	5.165^**^	−3.324^**^	0.526
total body fat mass (kg)	13.41 ± 5.7	16.98 ± 9.4	3.650^***^	21.17 ± 9.54	15.47 ± 8.92	13.711^***^	1.964	3.138^**^
Trunk fat mass (kg)	6.73 ± 3.44	9.51 ± 5.71	4.674^***^	12.01 ± 5.63	8.59 ± 5.48	17.917^***^	2.889^**^	3.098^**^
Center arm fat mass (kg)	0.55 ± 0.22	0.67 ± 0.43	2.863^**^	0.82 ± 0.43	0.61 ± 0.41	9.359^***^	1.368	2.622^*^
Right arm fat mass (kg)	0.58 ± 0.24	0.71 ± 0.43	3.145^**^	0.87 ± 0.44	0.65 ± 0.4	10.082^***^	1.669	2.637^**^
Center leg fat mass (kg)	2.75 ± 0.95	3.04 ± 1.47	1.924	3.67 ± 1.57	2.81 ± 1.36	8.161^***^	0.438	3.031^**^
Right leg fat mass (kg)	2.82 ± 0.99	3.14 ± 1.51	1.999^*^	3.77 ± 1.61	2.91 ± 1.4	8.01^***^	0.54	2.976^**^
Total body fat rate (%)	19.28 ± 6.83	24.05 ± 9.82	4.468^***^	28.28 ± 8.82	22.52 ± 9.75	16.099^***^	2.756^**^	3.024^**^
Trunk fat rate (%)	17.68 ± 7.53	25.57 ± 12.45	6.090^***^	30.73 ± 11.91	23.71 ± 12.16	24.63^***^	4.233^***^	2.903^**^
Center arm fat rate (%)	17.56 ± 6.72	20.65 ± 11.85	2.554^*^	25.15 ± 11.71	19.03 ± 11.52	8.394^***^	1.109	2.647^**^
Right arm fat rate (%)	17.46 ± 7.12	20.08 ± 11.21	2.215^*^	24.27 ± 11.25	18.57 ± 10.85	7.143^**^	0.865	2.602^*^
Center leg fat rate (%)	22.07 ± 6.76	23.57 ± 9.55	1.440	26.96 ± 9.1	22.34 ± 9.44	4.989^**^	0.25	2.466^*^
Right leg fat rate (%)	22.38 ± 6.84	23.85 ± 9.59	1.406	27.26 ± 9.27	22.62 ± 9.44	4.934^**^	0.221	2.469^*^
Total muscle mass (kg)	52.17 ± 8.92	48.53 ± 9.85	−3.041^**^	49.36 ± 12.05	48.22 ± 8.97	4.793^**^	−3.2^**^	0.502
Trunk muscle mass (kg)	28.82 ± 4.02	25.38 ± 4.73	−6.158^***^	25.82 ± 5.96	25.2 ± 4.21	19.166^***^	−6.383^***^	0.56
Center arm muscle mass (kg)	2.44 ± 0.66	2.31 ± 0.66	−1.554	2.25 ± 0.66	2.32 ± 0.65	1.369	−1.216	−0.575
Right arm muscle mass (kg)	2.59 ± 0.67	2.4 ± 1.25	−1.457	2.27 ± 0.72	2.43 ± 1.38	1.371	−1.005	−0.64
Center leg muscle mass (kg)	9.09 ± 1.93	9.2 ± 2.02	0.445	9.47 ± 2.41	9.09 ± 1.84	0.575	0.039	0.957
Right leg muscle mass (kg)	9.25 ± 2.06	9.27 ± 2.01	0.045	9.52 ± 2.44	9.16 ± 1.83	0.401	−0.312	0.791
Total skeletal muscle mass (kg)	29.73 ± 5.4	27.53 ± 5.96	−3.041^**^	28.03 ± 7.29	27.34 ± 5.42	4.793^**^	−3.199^**^	0.501
Trunk skeletal muscle mass (kg)	8.63 ± 1.25	6.73 ± 1.5	−10.857^***^	6.89 ± 1.86	6.66 ± 1.34	58.427^***^	−10.995^***^	0.665
Upper limb skeletal muscle mass (kg)	5.53 ± 1.45	5.18 ± 2.03	−1.552	4.98 ± 1.52	5.23 ± 2.17	1.469	−1.131	−0.638
Lower limb skeletal muscle mass (kg)	15.59 ± 3.38	15.69 ± 3.41	0.242	16.15 ± 4.12	15.51 ± 3.11	0.467	−0.14	0.817
ASM (kg/m^2^)	7.06 ± 1.26	6.95 ± 1.23	−0.694	6.79 ± 1.29	6.99 ± 1.19	0.565	−0.335	−0.816
Total body water (kg)	38.3 ± 6.65	33.93 ± 7.08	−5.005^***^	33.74 ± 7.82	33.98 ± 6.82	12.488^***^	−4.654^***^	−0.17
Mineral (kg)	2.88 ± 0.39	2.8 ± 0.48	−1.409	2.87 ± 0.56	2.76 ± 0.43	1.791	−1.892	1.16
Protein (kg)	13.57 ± 2.32	14.61 ± 4.42	2.355^***^	15.61 ± 5.29	14.24 ± 4.02	4.561^*^	1.465	1.38
Basal metabolic rate (kcal)	1,592.68 ± 253.33	1,483.57 ± 277.17	−3.228^***^	1,540.36 ± 337.94	1,463.23 ± 250.84	6.289^**^	−3.73^***^	1.217

*BMI, body mass index; FFM, fat free mass; ASM, appendicular skeletal muscle mass*.

* *P < 0.05*,

** *P < 0.01*,

*** *P < 0.001, statistically significant values are highlighted in bold*.

Most fat parameters (total body fat mass, trunk fat mass, total body fat rate and trunk fate rate, *p* < 0.001; left arm fat mass and right arm fat mass, *p* < 0.01; left arm fat rate and right arm fat rate, *p* < 0.05) of patients with WD was significantly higher than HC. While no statistically significant differences in the fat rate of legs have been found between patients with WD and HC.

On contrary, total muscle mass, total skeletal muscle mass, trunk muscle mass, and trunk skeletal muscle mass of patients with WD were significantly lower than HC (total muscle mass and total skeletal muscle mass, *p* < 0.01; trunk muscle mass, and trunk skeletal muscle mass, *p* < 0.001). However, there was no statistically significant difference has been found in the muscle mass or skeletal muscle mass of limbs between these two groups.

### The Characteristics of Anthropometry Parameters of Patients With WD

Table 2 shows the differences in anthropometry parameters between H-subtype WD patients and N-subtype WD patients. No statistically significant difference was found in BMI between these two groups.

**Table 2:** The characteristics of anthropometry parameters of patients with WD.

	**H subtype**	**N subtype**	***T*** **-value**
	**(***n*** = 35)**	**(***n*** = 95)**	
Height (m)	1.75 ± 0.09	1.71 ± 0.08	1.998^*^
Weight (kg)	73.18 ± 18.5	66.24 ± 13.46	1.792
BMI (kg/m^2^)	23.7 ± 5.04	22.43 ± 3.83	1.336
Waist circumference (cm)	84.99 ± 14.1	82.99 ± 10.34	0.769
hip circumference (cm)	99.55 ± 9.79	94.38 ± 12.17	2.258^*^
WHR	0.86 ± 0.08	0.96 ± 0.83	−0.732
TST (cm)	18.25 ± 7.68	15.06 ± 8.84	1.89
SST (cm)	18.82 ± 7.02	16.87 ± 6.77	1.438
biceps circumference (cm)	26.47 ± 4.58	25.77 ± 3.06	0.839
MAMC (cm)	24.28 ± 3.52	23.78 ± 2.48	0.766
calf circumference (cm)	37.07 ± 4.08	35.99 ± 3.86	1.392

*WHR, waist-to-hip ratio; TST, triceps skinfold thickness; SST, subscapular skinfold thickness; MAMC, mid-arm muscle circumference*.

* *P < 0.05*,

^**^P < 0.01,

^***^P < 0.001, H subtype compared with N subtype. Statistically significant values are highlighted in bold.

Most anthropometry parameters (Waist circumference, triceps skinfold thickness, subscapular skinfold thickness, mid-arm muscle circumference and calf circumference) of H-subtype WD patients were higher than N-subtype WD patients, but these differences had no statistical significance. Only the hip circumferences of H-subtype WD patients were significantly higher than N-subtype WD patients (*t* = 2.258, *p* < 0.05). However, the waist-to-hip ratio of H-subtype WD patients (0.86 ± 0.08) was lower than N-subtype WD patients (0.96 ± 0.83).

### Nutritional Status of WD Patients and the Characteristic of Parameters Among Patients of WD With and Without High Nutritional Risk

Table 3 shows the nutritional status of WD patients. Those patients who scored >3 points on the NRS2002 scale were defined as high nutritional risk. Patients with BMI ≥25 were defined as overweight subjects. Men with a body fat rate >21.4% and women with a body fat rate >29% were defined as having a high body fat rate (14).

**Table 3:** The nutritional status of patients with WD.

	**H-subtype**	**N-subtype**	***X^2^***	***P*** **-value**
	**(***n*** = 34)**	**(***n*** = 95)**		
**NRS2002**				
Normal nutritional status	26 (76.5%)	80 (84.2%)	1.024	0.312
High nutritional risk	8 (23.5%)	15 (15.8%)		
**BMI (overweight)**				
Normal weight	22 (64.7%)	74 (77.9%)	2.288	0.13
overweight	12 (35.3%)	21 (22.1%)		
**Body fat rate**				
Normal body fat rate	12 (35.3)	55 (57.9)	5.123	**0.024**
High body fat rate	22 (64.7%)	40 (42.1%)		

*Patients who scored >3 points on NRS2002 scale were defined as high nutritional risk. Patients with BMI ≥25 were defined as overweight. Men with a body fat rate >21.6% and women with a body fat rate >30 were defined as having a high body fat percentage. Statistically significant values are highlighted in bold*.

Additionally, 43.41% (56/129) of the patients with WD were found to have abnormal nutritional status. The prevalence of high-nutritional risk and overweight in WD patients was 17.83% (23 of 129) and 25.58% (33 of 129), respectively. No significant differences in the prevalence of high nutritional risk between H-subtype WD patients and N-subtype WD patients. The incidence of having a high body fat rate in patients with WD was 48.06%, and H-subtype WD patients were more likely to have a high body fat than N-subtype WD patients (*x*^2^ = 5.123, *p* < 0.05). A high body fat rate was more common (*x*^2^ = 10.829, *p* < 0.01) in female patients with WD (30 of 44) than male patients with WD (32 of 53).

### Characteristics of Serum Biochemical Biomarkers and Body Composition of Patients With a High Nutritional Risk

Table 4 shows essential parameters from serum biochemical test, blood routine examination (blood RT) and major body composition parameters among overweight WD patients and WD patients with high nutritional risk and WD patients with normal nutritional status. Compare with patients with high nutritional risk, the ratio of large platelets, alkaline phosphatase, BMR (*p* < 0.05), creatinine, trunk fat rate (*p* < 0.01), and ASM (*p* < 0.001) were significantly higher in patients without nutritional risk (*p* < 0.001).

**Table 4:** The characteristics of nutritional status among WD patients with and without high nutritional risk.

	**High nutritional risk** **(***n*** = 23)**	**Normal nutritional status** **(***n*** = 73)**	**Overweight** **(***n*** = 33)**	***F*** **-value**	***T*** **-value (High nutritional risk vs. Normal nutritional status)**	***T*** **-value (High nutritional risk vs. overweight)**	***T*** **-value (Normal nutritional status vs. overweight)**
**Blood routine examination**					
Red blood cells (× 10^12^/L)	4.37 ± 0.37	6.29 ± 13.97	4.67 ± 0.51	0.378	−0.626	−2.173^*^	0.604
Leukocytes (× 10^9^/L)	5.09 ± 2.00	5.00 ± 1.75	5.17 ± 1.71	0.086	0.189	−0.149	−0.417
Hemoglobin (g/L)	128.00 ± 13.38	134.00 ± 18.13	137.15 ± 15.23	1.809	−1.399	−2.176^*^	−0.795
Platelets (× 10^9^/L)	193.71 ± 114.31	175.61 ± 96.18	196.00 ± 120.43	0.471	0.718	−0.067	−0.861
Macro platelet ratio (%)	36.77 ± 7.07	32.26 ± 7.02	30.65 ± 7.22	4.734^*^	2.547^*^	2.943^*^	0.990
**Serum biochemical test**					
Total bilirubin (μmol/L)	13.67 ± 8.00	15.24 ± 9.59	12.59 ± 5.54	0.933	−0.599	0.518	1.312
Glutathione aminotransferase (U/L)	28.69 ± 26.48	34.06 ± 27.76	38.35 ± 28.30	0.608	−0.692	−1.101	−0.654
Glutathione transaminase (U/L)	24.63 ± 13.64	26.72 ± 11.12	29.43 ± 18.31	0.661	−0.634	−0.904	−0.700
Monoamine oxidase (U/L)	4.65 ± 1.70	4.51 ± 1.88	5.76 ± 2.01	5.230^**^	0.337	−2.163^*^	−3.125^**^
Creatinine (μmol/L)	61.78 ± 12.22	73.96 ± 22.67	70.00 ± 13.73	3.562^*^	−3.298^**^	−2.304^*^	1.105
Amylase	105.26 ± 80.42	90.79 ± 31.92	74.07 ± 22.13	3.726^*^	0.843	1.813	2.725^*^
Total protein	64.82 ± 4.22	65.17 ± 4.68	63.26 ± 5.84	1.663	−0.315	1.073	1.766
Albumin	41.45 ± 4.01	41.99 ± 4.44	40.12 ± 3.92	2.123	−0.510	1.213	2.039^*^
Globulin	23.28 ± 4.30	23.19 ± 4.53	23.15 ± 4.38	0.007	0.089	0.112	0.041
Albumin/ Globulin ratio	1.85 ± 0.41	1.90 ± 0.50	1.81 ± 0.42	0.5	−0.423	0.422	0.963
Fasting glucose (mmol/L)	5.03 ± 0.77	4.99 ± 0.79	5.15 ± 0.48	0.578	0.245	−0.693	−1.094
Glycosylated serum proteins (mmol/L)	1.89 ± 0.18	1.90 ± 0.26	1.79 ± 0.20	3.064	−0.127	2.148^*^	2.316^*^
Triglycerides (mmol/L)	0.89 ± 0.35	1.01 ± 0.52	1.13 ± 0.67	1.298	−1.028	−1.686	−0.958
Total cholesterol (mmol/L)	3.96 ± 0.89	4.28 ± 0.96	4.17 ± 1.02	0.982	−1.417	−0.796	0.546
Low density lipoprotein cholesterol (mmol/L)	2.36 ± 0.93	2.71 ± 0.80	2.63 ± 0.83	1.478	−1.717	−1.129	0.439
Alkaline phosphatase (U/L)	104.74 ± 46.94	87.03 ± 28.83	87.22 ± 34.40	2.54	2.183^*^	1.614	−0.029
Cholinesterase (U/L)	4,726.78 ± 1,292.50	5,252.74 ± 1,362.34	5,477.67 ± 1,401.34	2.106	−1.634	−2.036^*^	−0.780
Ceruloplasmin (mg/L)	58.60 ± 42.81	50.59 ± 22.95	59.25 ± 34.32	1.206	0.859	−0.063	−1.527
Copper oxidase (OD)	0.06 ± 0.05	0.06 ± 0.03	0.07 ± 0.05	2.234	1.342	−0.355	−2.146^*^
Serum copper (μmol/L)	3.01 ± 1.70	2.68 ± 1.93	3.28 ± 2.47	1.025	0.757	−0.436	−1.353
25(OH) vitamin D (ng/mL)	16.00 ± 6.87	17.93 ± 8.17	17.23 ± 6.67	0.574	−1.004	−0.658	0.434
**Bioelectrical parameters**					
Total body fat rate (%)	18.17 ± 0.08	21.68 ± 7.91	33.30 ± 8.54	30.354^***^	−1.835	−6.588^***^	−6.833^***^
Trunk fat rate (%)	7.19 ± 6.44	12.59 ± 9.8	22.69 ± 17.50	13.218^***^	−3.058^**^	−4.655^***^	−3.102^**^
ASM (kg/m^2^)	6.03 ± 0.71	6.8 ± 0.98	7.9 ± 1.39	22.684^***^	−3.517^***^	−5.921^***^	−4.679^***^
Mineral (kg)	2.43 ± 0.42	2.71 ± 0.33	3.24 ± 0.46	34.061^***^	−2.911^**^	−6.718^***^	−6.692^***^
Basal metabolic rate (kcal)	1,293.77 ± 218.75	1,424.12 ± 192.23	1,747.38 ± 292.43	33.048^***^	−2.743^*^	−6.305^***^	−5.808^***^

*ASM, appendicular skeletal muscle mass*.

* *P < 0.05*,

** *P < 0.01*,

*** *P < 0.001, statistically significant values are highlighted in bold*.

Among these three groups, significant differences can be found in the ratio of large platelets (*f* = 4.734, *p* < 0.05, overweight < normal nutritional status < high nutritional risk), monoamine oxidase (*f* = 5.230, *p* < 0.01, normal nutritional status < high nutritional risk < overweight), creatinine (*f* = 3.562, *p* < 0.05, high nutritional risk < overweight < normal nutritional status), amylase (*f* = 3.726, *p* < 0.05, overweight < normal nutritional status < high nutritional risk), total body fat rate (*f* = 30.354, *p* < 0.001, high nutritional risk < NNS < overweight), trunk fat rate (*f* = 13.218, *p* < 0.001, high nutritional risk < normal nutritional status < overweight), ASM (*f* = 22.684, *p* < 0.001, high nutritional risk < normal nutritional status < overweight), mineral (*f* = 34.061, *p* < 0.001, high nutritional risk < normal nutritional status < overweight) and BMR (*f* = 33.048, *p* < 0.001, high nutritional risk < normal nutritional status < overweight).

There are no statistically significant differences that have been found in red blood cells, hemoglobin, glutathione aminotransferase, glutathione transaminase total protein, albumin, globulin, glucose, 25(OH) vitamin D and lipid between patients with high nutritional risk and patients with normal status.

Compared with patients with a high nutritional risk, cholinesterase was higher in patients with a normal nutritional status (high nutritional risk: 4,726.78 ± 1,292.50, normal nutritional status: 5,252.74 ± 1,362.34), however, there was no statistically significant difference between these two groups (*t* = −1.634, *p* > 0.05). Less than 4,400 U/L was defined as having a low concentration of cholinesterase, and patients with a high nutritional risk tend to have a lower cholinesterase concentration (*x*^2^ = 4.227, *p* < 0.05).

### The Correlation of Nutritional Status and Treatment

Table 5 showed the correlation between nutritional status and treatment. The results showed that there is no significant difference in nutritional risk between before and after medication.

**Table 5:** The correlation of nutritional risk status and WD treatment.

	**Normal nutritional status**	**High nutritional risk**	***P***
	**(***n*** = 106)**	**(***n*** = 23)**	
**Chelators use**			
Before	13 (12.3%)	1 (4.3%)	0.242
After	93 (87.7%)	22(95.8%)	
**Zinc agent use**			
Before	14 (13.2%)	0	0.062
After	93 (87.7%)	22 (100%)	

### The Correlation of Nutritional Status and Severity

Table 6 showed the correlation of nutritional status and severity, including UWDRS total scores, UWDRS neurological scores, UWDRS hepatic scores and UWDRS mental scores. The results showed that there is no significant difference in UWDRS total scores, UWDRS neurological scores, UWDRS hepatic scores and UWDRS mental scores between patients with and without a nutritional risk.

**Table 6:** The correlation of nutritional risk status and severity.

	**Normal nutritional status**	**High nutritional risk**	***T***	***P***
UWDRS total score	24.91 ± 24.87	31.26 ± 26.26	1.100	0.273
UWDRS neurological score	19.05 ± 21.12	24.26 ± 23.57	1.051	0.295
UWDRS hepatic score	2.42 ± 2.62	3.65 ± 4.05	1.402	0.173
UWDRS mental score	3.44 ± 4.61	3.35 ± 3.88	0.093	0.926

## Discussion

### Major Findings

This study explored the characteristics of nutritional status of patients with WD, and there were four major findings. First, we detected the body composition of patients with WD by bioelectrical impedance measurements. We find that there are some differences in body composition among healthy individuals, patients with H-subtype and N-subtype, especially in fat rate and muscle mass. Second, we determined the anthropometric data of patients with WD and find that the height, hip circumference, waist-to-hip ratio among patients with H-subtype WD are higher than patients with N-subtype WD. Third, we investigated the abnormal nutritional status among patients with WD, and find that 17.83% of the patients have a high nutritional risk. Finally, the ratio of large platelets, monoamine oxidase, creatinine, amylase and cholinesterase can reflect different nutritional statuses, as well as trunk body fat rate, appendicular skeletal muscle mass, mineral and basal metabolic rate.

### Body Composition Factors Associated With Abnormal Body Composition in Patients With WD

While most previous studies have revealed that patients with chronic disease usually have a higher level of body fat and a lower level of muscle (11, 12, 15), we specifically explored the detailed characteristics of patients, to discover how it differs from other chronic diseases. Previous studies reported that patients with WD usually have a high body fat rate and a low muscle mass (16). However, these results cannot characterize the body composition of the different phenotypes of patients with WD comprehensively. In our study, we focus on the differences in body composition among healthy subjects, patients with H-subtype and N-subtype. At the same level of BMI, both trunk and appendicular body fat rates of patients with H-subtype were higher than healthy individuals. While patients with N-subtype only have a higher trunk body fat rate than healthy individuals, whereas there was no difference in appendicular body fat rate compared to healthy individuals. Additionally, the mass of muscle and skeletal muscle in both patients with H-subtype and N-subtype are lower than healthy individuals at the trunk part, while there are no differences at the appendicular section. Different distribution of skeletal muscle and body fat mass may be due to patients with N subtypes having neurological or movement disorders compared to patients with H subtypes.

### Anthropometric Factors Related to Abnormal Nutritional Status in Patients With WD

Anthropometric measurements are usually applied in the absence of biochemical conditions and suitable bioelectrical impedance instruments (17–19). Among these parameters, skinfold thickness can reflect the overall level of the fat rate at the section of the trunk and limbs (subscapular skinfold thickness and triceps skinfold thickness, respectively) (20). Additionally, the waist-to-hip ratio reflects the degree of central obesity, which can reveal the mortality risk of subjects (21). Generally, individuals with a high WHR (male> 0.9, female> 0.85) are defined as having an “apple shape,” which means tend to have more visceral fat than those who have a pear-shaped, hourglass figure with a lower WHR (22). Previous studies demonstrated that WHR is positively associated with the risk of insulin resistance, hypertension, hyperlipidemia, hypercholesterolemia and other cardiometabolic diseases (23–25). According to our study, we draw the following two inferences. First, no differences had been found in skinfold thickness between patients with H-subtype and N-subtype, which is inconsistent with the results of the previous body composition analysis. This means that anthropometric measurements are less stable and its results are not representative of the actual body composition of patients with WD. Second, most patients have a high WHR and patients with N-subtype are closer to an apple shape than patients with H-subtype. This means that although patients with N-subtype have a lower total fat percentage than B, it has more fat concentrated in the viscera. From nutritional status and comorbidity perspectives, our study suggests that patients with WD need a combined sports nutrition intervention project to control the visceral fat, which can attenuate their cardiovascular-related all-cause mortality and ameliorate their prognosis.

### The Relationship Between Nutritional Status and Blood Biomarkers

NRS2002 has a stronger capability in adult patients compared with Malnutrition Universal Screening Tool (MUST) and Patient-generated Subjective Global Assessment (PG-SGA) (26). Thus, it is recommended by the ESPEN guidelines to be a conventional nutritional risk screening tool among patients with chronic diseases (13). In this study, we used NRS2002 to screen the prevalence of malnutrition in patients with WD, and we find that the prevalence of having a high nutritional risk is 17.83%. Besides, 25.58% of the patients are overweight, thus the overall prevalence of abnormal nutritional status among patients with WD is 43.14%. Additionally, patients with H-subtype and N-subtype have the same opportunity to suffer from high nutritional risk. Notably, we find that a high body fat rate is more likely to occur in female patients and patients with H-subtype. Former studies had proved that exercise and nutritional interventions can recover the abnormal nutritional status (27).

We also find that the ratio of large platelets, monoamine oxidase, creatinine, amylase, and cholinesterase have strong correlations with the nutritional status of patients with WD. The ratio of large platelets can enhance the risk of acutely ischemic brain stroke and atherosclerosis, which might be caused by the over-functional-active platelets releasing more active substances (28). According to our study and the above report, patients with WD and a high nutritional risk may have a high risk of atherosclerosis and acute vascular events. Monoamine oxidase is frequently regarded as a parameter to assess the hepatic injury and it may influence the food intake of patients with WD through appetite regulation (29). Hsu et al. (30) reported that higher serum creatinine concentrations were associated with a lower relative risk for malnutrition-related death. In our study, overweight patients and patients with a high nutritional risk have lower serum creatinine concentrations, which match the above study. Su et al. (31) reported that higher serum amylase constantly occurred among patients with atrophic gastritis and it had a correlation with malnutrition. As early as 1989, Ollenschläger et al. (32) recommended cholinesterase as an indicator for nutritional assessment, and they pointed out that the diagnosis of malnutrition can be made when cholinesterase is lower than the normal range or reduced by 10%. According to our research, patients with a high nutritional risk are more likely to have a lower serum cholinesterase concentration.

### Limitations and Outlook

There are several limitations to this study. First, although NRS2002 is a well-validated screening tool for abnormal nutritional status, we found it may still not be enough to quantify the abnormal nutritional status for patients with WD. We will pay more attention to selecting or developing better nutrition screening tools specifically for patients with WD in the future. Second, although there are many conveniences testing body composition by BIA, like quick and comprehensive. However, the body composition values calculated by the data model are still less convincing and acceptable compared to Dual- energy X-ray absorptiometry, Computed Tomography and other equipment. Thirdly, this study is a cross-sectional observation focused on the abnormal nutritional status and its detailed characteristics of patients with WD. We have no idea about the correlations between the nutritional status and the prognosis. Longitudinal studies are required to investigate if nutritional status and body composition could reflect prognosis in WD patients, and which of these indexes of body composition and bloody parameters contribute to abnormal nutritional status and worse prognosis. Finally, the guidelines of exercise and nutrition intervention prescription for patients with WD are imperfect. We aim to develop the interventions of exercise and nutrition, and then explore the indications, contraindications, dosage, frequency, adverse reactions and other specific requirements, for earlier clinical application.

## Conclusion

In conclusion, both patients with H-subtype and N-subtype are prone to have an abnormal nutritional status. However, it is different in the form of abnormal nutritional status between patients with these two subtypes. Patients with N-subtype have a bigger waist-to-hip ratio and a more “apple-shaped” body, suggesting that patients with N-subtype are at higher risk of suffering from atherosclerosis and acute cardiovascular disease in the future. Among the blood test indicators, we also identified several markers that were associated with nutritional status, including the ratio of large platelets, monoamine oxidase, creatinine, amylase and cholinesterase. Nutritional status is not related to the severity and medication history. Longitudinal studies are required to investigate if nutritional status and body composition could reflect prognosis in WD patients, and which of these body composition indexes contribute to malnutrition and worse prognosis.

## Data Availability Statement

The raw data supporting the conclusions of this article will be made available by the authors, without undue reservation.

## Ethics Statement

The studies involving human participants were reviewed and approved by the Ethics Committee of Hefei Institutes of Physical Science, Chinese Academy of Sciences. Written informed consent to participate in this study was provided by the participants' legal guardian/next of kin.

## Author Contributions

HG: study concept and design, data validation, statistical analyses, writing—original draft preparation, and revising final manuscript. SW: data validation and proofreading manuscript. YJ, BS, SS, BL, YongsH, YongzH, LG, ZD, and YX: data acquisition. NC: proofreading manuscript. XW: conception and design of the study. ZM: study design and proofreading manuscript. YS: study design, data, acquisition, writing—review and editing and refinement, and supervision. All authors contributed to the article and approved the submitted version.

## Funding

This study was supported partly by grants 2020YFC2005600 from National Key R&D Program of China, 2020sjzd01 from Scientific Research Fund of Anhui University of Chinese Medicine and 1908085QH34 from Natural Science Foundation of Anhui Province for clinical diagnosis and research criteria checking. The funder had no role in study design, data collection and analysis, decision to publish or preparation of the manuscript.

## Conflict of Interest

The authors declare that the research was conducted in the absence of any commercial or financial relationships that could be construed as a potential conflict of interest.

## Publisher's Note

All claims expressed in this article are solely those of the authors and do not necessarily represent those of their affiliated organizations, or those of the publisher, the editors and the reviewers. Any product that may be evaluated in this article, or claim that may be made by its manufacturer, is not guaranteed or endorsed by the publisher.

## Abbreviations

WD: Wilson's disease
HC: healthy controls
H-subtype: hepatic subtype
N-subtype: neurological subtype
PD: Parkinson's disease
AD: Alzheimer's disease
NRS2002: nutritional risk screening 2002
MNA: mini nutritional assessment
MCI: mild cognitive impairment
BMI: body mass index
WHR: waist-to-hip ratio
MUST: Malnutrition Universal Screening Tool
PG-SGA: Patient-generated Subjective Global Assessment
UWDRS: Unified Wilson's Disease Rating Scale.
